# An Unusual Conformational Isomer of Verrucosidin Backbone from a Hydrothermal Vent Fungus, *Penicillium* sp. Y-50-10

**DOI:** 10.3390/md14080156

**Published:** 2016-08-18

**Authors:** Chengqian Pan, Yutong Shi, Bibi Nazia Auckloo, Xuegang Chen, Chen-Tung Arthur Chen, Xinyi Tao, Bin Wu

**Affiliations:** 1 Ocean College, Zhejiang University, Hangzhou 310058, China; 3100103262@zju.edu.cn (C.P.); 11434028@zju.edu.cn (Y.S.); naz22ia@hotmail.com (B.A.N.); chenxg83@gmail.com (X.C.); 2 Department of Oceanography, National Sun Yat-sen University, Kaohsiung 80424, Taiwan; ctchen@mail.nsysu.edu.tw; 3 State Key Laboratory of Bioreactor Engineering, East China University of Science and Technology, Shanghai 200237, China; xytao@ecust.edu.cn

**Keywords:** marine fungus, *Penicillium* sp., verrucosidin, conformational isomer, antibiotic

## Abstract

A new verrucosidin derivative, methyl isoverrucosidinol (**1**), was isolated from the marine fungus *Penicillium* sp. Y-50-10, dwelling in sulfur rich sediment in the Kueishantao hydrothermal vents off Taiwan. The structure was established by spectroscopic means including HRMS and 2D-NMR spectroscopic analysis. The absolute configuration was defined mainly by comparison of quantum chemical TDDFT calculated and experimental ECD spectra. Among hitherto known compounds with a verrucosidine backbone isolated from natural resource, compound **1** represents the first example of a new conformational isomer of its skeleton, exhibiting antibiotic activity against *Bacillus subtilis* with MIC value 32 μg/mL.

## 1. Introduction

Marine microorganisms have been under the spotlight of scientists during the last decade due to their tremendous abilities of generating exceptional and potent natural products [1,2]. In comparison to terrestrial environments, extreme marine environments bearing unique physico-chemical parameters such as high temperature, pressure, salinity or a deprivation from nutrition and dissolved oxygen gave rise to novel biologically active metabolites which can be further developed for the benefits of humanity [3]. It was proven that marine fungi can produce an assortment of secondary metabolites, protecting them from predators as well as acting as effective ways of communication between species [4,5]. As a consequence, these adaptation strategies which enable the production of distinctive compounds with new chemical structures can exhibit substantial bioactivities against pathogens [6].

**Figure 1:**
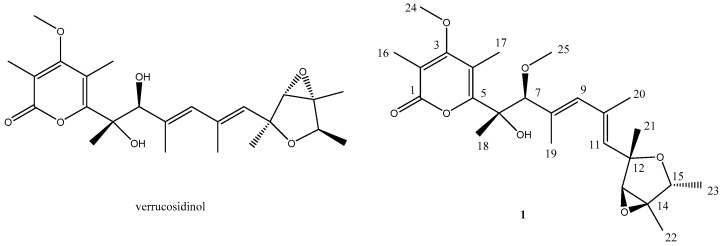
Compound **1** isolated from the extract of fungus *Penicillium* sp. Y-50-10 and verrucosidinol for comparison.

Verrucosidin, isolated from *Penicillium verrucosum* var. *cyclopium* [7,8,9,10], was regarded as a powerful neurotoxin (LD_50_ in mice, 4 mg/kg, i.p.). It is considered as a down-regulator of the *grp*78 gene leading to selective cell death under low glucose levels [11]. In this study, an unusual conformational isomer of verrucosidin backbone, methyl isoverrucosidinol (**1**) (Figure 1), was isolated from the fermentation of the hydrothermal vent fungi, *Penicillium* sp. Y-50-10. This paper describes the isolation, structure elucidation as well as the bioactivity of the isolated compound from that particular hydrothermal vent fungus.

## 2. Results

The MeOH extracts of the mycelia and the EtOAc extracts of broth were subjected to repeated column chromatography to purify compound **1**.

**Figure 2:**
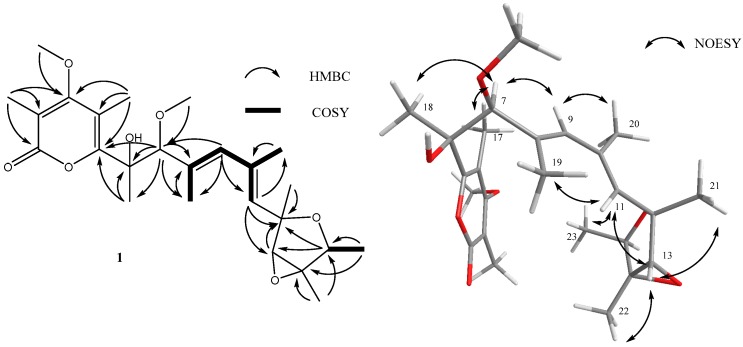
Key HMBC, ^1^H–^1^H COSY and key NOESY correlations of compound **1**.

Methyl isoverrucosidinol (**1**) was obtained as light yellow oil. The molecular formula C_25_H_36_O_7_ was determined by analysis of the HR-TOF-MS data (Figure S8). The formula was supported by the ^13^C-NMR data, which indicated 11 degrees of unsaturation. The NMR data (Table 1, Figures S1–S7) of compound **1** were similar to those of α-pyrone-type polyketides, verrucosidinols analogues [12,13]. These molecules contain two distinct domains, a dicyclic 3,6-dioxabicyclo[3.1.0]hexane and a α-pyrone ring, connected by a olefinic chain [12,13]. The ^13^C NMR spectrum showed the presence of eight signals for the α-pyrone ring domain including a 2*H*-pyran-2-one basic skeleton and two methyls and a methoxyl, and seven signals for dicyclic fused rings including a 3,6-dioxabicyclo[3.1.0]hexane skeleton and three methys, with the remaining nine resonances corresponding to an olefinic chain moiety. Fungal derived α-pyrone-type polyketides comprise mainly of two groups, verrucosidinol analogues [12,13] and aurovertin analogues [14]. The main difference between these two skeletons lies at three methyls on the olefinic chain for the verrucosidinol skeleton, whereas the olefinic chain in aurovertin skeleton possesses no methyl group. When compared to the NMR data of the polyketide verrucosidinol [12,13], compound **1** showed similar chemical shifts of the two main substructural domains. The presence of the characteristic ^13^C NMR methyl signals at δ_C_ 22.1, 13.3 and 17.6 on the olefinic chain ruled out the possibility of the presence of the aurovertin skeleton. However, according to the NMR data, the substructure of the olefinic chain was less similar to verrucosidinol. In order to elucidate the planar structure of the new compound, detailed 1 D and 2 D NMR experiments were carried out. The ^13^C NMR chemical shifts of five downfield signals C-1 (δ_C_ 166.0), C-2 (δ_C_ 109.4), C-3 (δ_C_ 170.4), C-4 (δ_C_ 112.3) and C-5 (δ_C_ 160.5) were in close agreement to those for an α-pyrone unit. The quartet methine protons of H-15 (δ_H_ 4.05, *J* = 6.8 Hz), the doublet methyl protons of H-23 (δ_H_ 1.18, *J* = 6.8 Hz), and the ^1^H–^1^H COSY signal (Figure 2) between them suggested a connection between C-15 and C-23. The correlations from the methyl protons of H-23 to C-14 (δ_C_ 67.4) and C-15 (δ_C_ 76.9), from the methine proton of H-15 (δ_H_ 4.05) to C-12 (δ_C_ 80.0) and C-13 (δ_C_ 67.3), from the methyl protons of H-22 (δ_H_ 1.47) to C-14 and C-15, from the methine proton H-13 (δ_H_ 3.55) to C-12, thereby establishing the existence of a tetrahydrofuran ring moiety in **1**. The HMBC correlations from the methyl protons of H-18 (δ_H_ 1.45) to C-6 (δ_C_ 78.7) and C-5 (δ_C_ 160.5), from the methine proton of H-7 (δ_H_ 3.91) to C-6, C-8 (δ_C_ 132.7), C-18 (δ_C_ 22.1) and C-19 (δ_C_ 13.3), from the methyl protons H-19 (δ_H_ 1.79) to C-8, from the methine proton H-9 (δ_H_ 5.77) to C-7, C-19, and C-11 (δ_C_ 132.0), from the methyl protons H-20 (δ_H_ 1.89) to C-10 (δ_C_ 135.1), from the methine proton of H-11 (δ_H_ 5.45) to C-20 (δ_C_ 17.6), C-12 (δ_C_ 80.0) and C-13 (δ_C_ 67.3), from the methyl protons H-21 (δ_H_ 1.37) to C-12 completed the assignment of a heptadiene moiety. The ^1^H–^1^H COSY revealed correlations from H-9 to H-19, from H-9 to H-20 and from H-20 to H-11, which confirmed this deduction. The methoxy group was deduced to be attached to C-7, which was inferred from the cross peak between H-25 (δ_H_ 3.25) and C-7 in the HMBC spectrum of **1** (Figure 2). The HMBC correlations from methine proton of H-7 to C-5 and from the methyl protons H-18 to C-5 revealed linkage between the heptadiene moiety and the α-pyrone moiety. The long range correlation cross peak from the methine proton H-11 to C-12 and C-13 connected the heptadiene moiety and the tetrahydrofuran ring moiety. From the above analysis, the planar structure of **1** was elucidated as a methylated derivative of verrucosidinol drawn in Figure 1. The geometry of C_8_=C_9_ and C_10_=C_11_ were confirmed to be *E* by the analysis of NOE observation. The NOESY enhancements between H-11 and H-13, H-13 and H-22, and H-11 and H-23 supported the orientations of these groups in tetrahydrofuran ring (Figure 2), showing the same results as verrucosidinol in literature [12,13]. The NOESY enhancements between H-7 and H-17, H-18, H-9, and the weak enhancement between H-18 and H-9 determined their steric configuration respectively as shown in Figure 2. Since the rotation of C-7 was hindered by the contact of pyran-2-one and the OH or OMe at C-7, the relative configuration of C-7 was achieved with NOESY experiments by Lixin Zhang’s groups [12]. The results in this study confirmed the relative configuration of C-7 (Figure 2).

However, key NOESY enhancements between H-9 and Me-20, H-11 and Me-19, allowed the assignment of a new conformational isomerization between double bond C_8_=C_9_ and C_10_=C_11_. This is the first example of discovering such an unusual conformational isomer of verrucosidin skeleton. 

How is the new conformation of C_8_=C_9_–C_10_=C_11_ immobilized? Although these two isomers have a same C=C–C=C architecture, there exists a basic difference, which is the steric location of Me-19 and Me-20.

It is known that the methyl group is not bulky enough to suppress the rotation of the single bond in the olefin chain. However, the principal barriers to rotation about the olefin chain in compound **1** are the substituent bulky ring system attached on each side of the C_8_=C_9_–C_10_=C_11_, which formed a U shape of molecule (Figure 2). Obviously, close contacts occur with two ring systems of 2*H*-pyran-2-one and dicyclic fused 3,6-dioxabicyclo[3.1.0]hexane, preventing the free rotation. The structures, therefore, fall into two classes: those in which the Me-19, Me-20 are on one side and those which are on different side. As far as the steric effect on rotation is concerned, the close contacts make the existence of two isomers and make it extremely unlikely that two isomers transform in ordinary condition.

**Figure 3:**
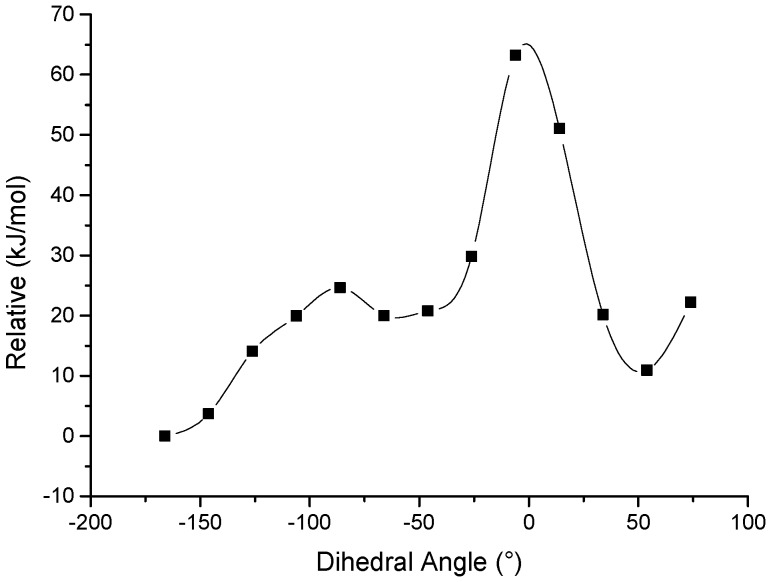
The potential energy surface scan by different dihedrals between C_8_=C_9_ and C_10_=C_11_.

In order to confirm the stability of the conformation, rotation barrier was calculated by Gaussian 09 scan program. 13 different transition states were calculated by different dihedrals between C_8_=C_9_ and C_10_=C_11_ from −166° to 80°. Configuration optimizations were done by employing density functional theory (DFT) B3LYP method (Tables S1 and S2). Same combined basis set was used, in which C, H and O atoms were described by 6-31+G(d,p) basis set. Frequency analysis was done at the same level of theory to verify that these optimized structures are real minima on the potential energy surface. The calculated results were elucidated as drawn in Figure 3. It was shown that two low-lying isomers are normal verrucosidin skeleton (0 kJ/mol) and compound **1** (10 kJ/mol), the high rotation barrier of at least 68 kJ/mol between two low-lying isomers prove that two conformational isomers could not transform spontaneously under room temperature (Figure 3). The remaining isomers are either kinetically unstable with low barriers or energetically very high lying.

**Figure 4:**
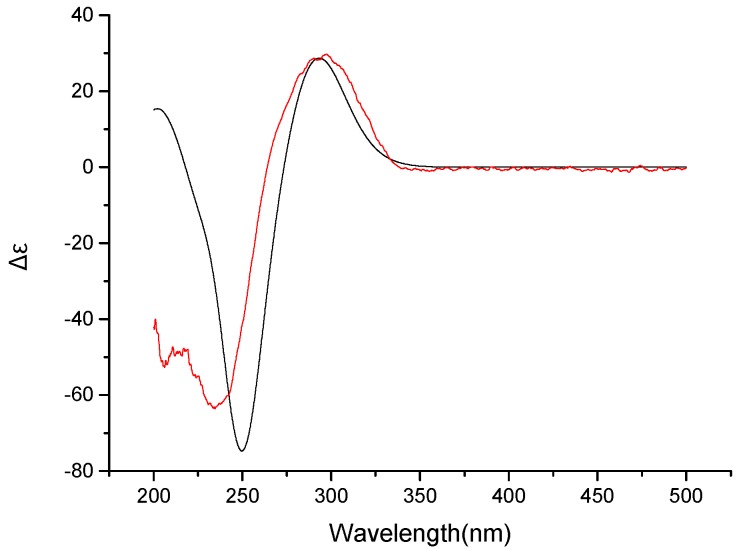
B3LYP/6-31+G(d,p) calculated ECD spectra of 6*S*,7*S*,12*S*,13*S*,14*R*,15*R* (**black**) and the experimental ECD spectrum (**red**) (σ = 0.25 eV) for compound **1**.

The absolute configurations of **1** were determined by simulation of the ECD spectrum. The minimum energy geometries of two conformers were optimized using DFT at the B3LYP/6-31+G(d,p) level in the gas phase by the GAUSSIAN 09. Then the ECD spectra Calculation were simulated using the time-dependent density functional theory (TDDFT) employing the B3LYP functional at the B3LYP/6-31+G(d,p) level in methanol. The calculated ECD curves were drawn by using SpecDis with a σ of 0.25 eV (Figure S9). The calculated ECD spectrum for 6*S*,7*S*,12*S*,13*S*,14*R*,15*R* showed a negative Cotton effect at 250 nm and a positive Cotton effect at 300 nm which is identical to the experimental ECD spectra. Therefore, the absolute configurations of **1** were determined as 6*S*,7*S*,12*S*,13*S*,14*R*,15*R* (Figure 4). Compound **1** was named as methyl isoverrucosidinol.

In order to rule out the possibility that the methoxy originated from the methanol used in the extraction or methanol/silica in the purification, a small scale experiment was conducted. *Penicillium* sp. Y-50-10 was cultured under static conditions in Erlenmeyer flasks for one week. The medium was extracted with EtOAc. The EtOAc extract was evaporated and dissolved in acetonitrile. The MS result showed that compound **1** presents without methanol in the extraction, purification and measurement (Figure S10).

### Biological Activity

The antibacterial activities of the isolated compound were evaluated against three terrestrial pathogenic bacteria, *Escherichia coli*, *Staphylococcus aureus* and *Bacillus subtilis*. Compound **1** exhibited weak antibiotic activity with MIC values of around 32 μg/mL against *B. subtilis*. However, the MIC values of the compound obtained against *E. coli* and *S. aureus* were greater than 64 μg/mL and thus were considered as “negligible activity”. Tetracycline was used as a positive control against *E. coli*, *S. aureus* and *B. subtilis* which showed MIC values of 2, 2, 1 μg/mL, respectively.

## 3. Materials and Methods

### 3.1. General Experimental Procedures

Optical rotations were measured in MeOH on a Perkin-Elmer-341 polarimeter. The IR spectra were run on a NicoletAvatar-360FT-IR spectrometer. ^1^H NMR (500 MHz) and ^13^C NMR (125 MHz) spectra were measured at 25 °C on a Bruker AVANCE DMX 500 NMR spectrometer with TMS as internal standard. CD spectra were measured on a JASCO J-715 (JASCO) spectropolarimeter. UV spectra were also recorded in MeOH on a Shimadzu UV2550. ESIMS were recorded on an Agilent 6460 Triple Quad LCMS. Prep. HPLC was performed on a ChuangXinTongHeng system equipped with a Venusil MP-C18 column (10 mm × 250 mm, Agela Technologies, Tianjin, China).

### 3.2. Isolation and Cultivation of the Fungal Strain

*Penicillium* sp. Y-50-10 was isolated from the Sulfur rich sediment, Kueishantao, Taiwan in May 2014. The fungus was cultured under static conditions at 28 °C in 500 mL Erlenmeyer flasks containing 200 mL of liquid culture medium composed of glucose (10.0 g/L), potato dextrose agar (PDA) (20.0 g/L), and seawater. After 3 weeks of cultivation, 5 L of whole broth was filtered to separate the thallus. After fermentation, the medium was extracted with EtOAc. The fungus was classified as *Penicillium* sp. on the basis of the ITS 18S fragment.

### 3.3. Extraction and Isolation

5 L of the fungal culture broth was filtered. The air-dried thallus part was extracted at room temperature with MeOH. The residues were dissolved in H_2_O (250 mL) and extracted with EtOAc (3 × 500 mL). The broth (5 L) was extracted with EtOAc (2 × 5 L). Both were combined and concentrated to give an organic extract (0.69 g). The extract was subjected to liquid chromatography over a silica gel column using a gradient elution of petroleum ether (PE)–EtOAc–MeOH (PE:EtOAc = 1:0, 20:1, 10:1, 5:1, 2:1, 1:1, 0:1 & MeOH) to give several fractions. Seven main fractions were obtained which was confirmed by TLC. Every fraction was dissolved in methanol and centrifuged at 12,000 rpm for 10 min. The supernatant was collected and subjected to preparative HPLC (10.0 mL/min, UV = 210 nm), using MeOH:H_2_O as eluent. Fraction 4 was separated by preparative HPLC (MeOH:H_2_O = 50:50, 10 mL/min) to afford compound **1** (1.4 mg, t_R_ 26.0 min).

### 3.4. Computation Section

The geometry was optimized starting from initial conformations, with DFT calculations at the B3LYP/6-31+G(d,p) level using the Gaussian09 program. Time-dependent DFT calculations were performed on the lowest-energy conformations for each configuration using 30 excited states and under the CH_3_OH solution. ECD spectra were generated using the program SpecDis by applying a Gaussian band shape with 0.3 eV width, from dipole-length rotational strengths [15,16].

Methyl isoverrucosidinol (**1**): light yellow oil; ${\left[\alpha \right]}_{D}^{20}$ −1.23 (*c* 0.1, CH_3_OH); UV/vis (CH_3_OH) λ_max_ (logε) 200 (4.23), 232 (3.95), 297 (3.69); IR ν_max_ 3415, 2917, 2849, 1700, 1684, 1559, 1457, 1379, 1352, and 1090 cm^−1^; CD (c 4.5 × 10^−4^ mol/L, CH_3_OH) λ_max_ (Δε): 214 (−48.5), 236 (−63.5), 295 (−28.8), 340 (−0.3); ^1^H and ^13^C NMR, Table 1.; HRESIMS *m*/*z* 449.2544 [M + H]^+^ (calcd. for C_25_H_37_O_7_^+^, 449.2539).

### 3.5. Biological Assays

Antibacterial activities were evaluated by the conventional broth dilution assay [17]. Three bacterial strains, *E. coli* [CMCC (B) 44102], *S. aureus* [CMCC (B) 26003], *B. subtilis* [CMCC (B) 63501], were used, and tetracycline was used as positive control.

## 4. Conclusions

Due to the duplication in the isolation of secondary metabolites from terrestrial sources, the marine world can be considered as the sole source of novel compounds with beneficial applications in the medical sector. Among hitherto known compounds with a verrucosidine backbone found in nature, methyl isoverrucosidinol (**1**) represents the first example of a new conformational isomer of its skeleton. The relationship between conformation and bioactivity of natural products is worth exploring further. This study also proved that marine microorganisms, especially the ones thriving in extreme conditions have the capacities of generating new and unique natural products. Therefore, more work should be done in order to discover novel potent bioactive compounds which could further unveil the secrets of combatting infectious diseases forever.

## Figures and Tables

**Table 1:** ^1^H NMR data (500 MHz, δ in ppm, *J* in Hz), ^13^C NMR data (125 MHz, δ in ppm) and for **1**. Taken in CD_3_OD.

Carbon Number	δ_C_, Type ^a^	δ_H_ (*J* in Hz)	Carbon Number	δ_C_, Type ^a^	δ_H_ (*J* in Hz)
1	166.0, C		14	67.4, C	
2	109.4, C		15	76.9, CH	4.05, q (6.8)
3	170.4, C		16	8.9, CH_3_	2.00, s
4	112.3, C		17	8.8, CH_3_	2.25, s
5	160.5, C		18	22.1, CH_3_	1.45, s
6	78.7, C		19	13.3, CH_3_	1.79, s
7	90.6, CH	3.91, s	20	17.6, CH_3_	1.89, s
8	132.7, C		21	20.8, CH_3_	1.37, s
9	135.3, CH	5.77, s	22	12.5, CH_3_	1.47, s
10	135.1, C		23	17.8, CH_3_	1.18, d (6.8)
11	132.0, CH	5.45, s	24	59.7, CH_3_	3.84, s
12	80.0, C		25	55.7, CH_3_	3.25, s
13	67.3, CH	3.55, s			

^a^ Multiplicities inferred from DEPT and HMQC experiments.

## Supplementary Materials

The following are available online at http://www.mdpi.com/1660-3397/14/8/156/s1, Figure S1: ^1^H NMR in CD_3_OD for compound **1**, Figure S2: ^13^C NMR in CD_3_OD for compound **1**, Figure S3: DEPT in CD_3_OD for compound **1**, Figure S4: COSY in CD_3_OD for compound **1**, Figure S5: HMQC in CD_3_OD for compound **1**, Figure S6: HMBC in CD_3_OD for compound **1**, Figure S7: NOESY in CD_3_OD for compound **1**, Figure S8: HRESIMS for compound **1**, Figure S9: B3LYP/6-31+G(d,p) calculated ECD data for conformations of compound **1**, Figure S10: MS data (TIC and XIC [M + H]^+^ 449 Da) for acetonitrile dissolved *Penicillium* sp. Y-50-10 extract, Table S1: Gibbs Free Energy (Hartree/Particle) of compound **1**, Table S2: Total Energy (Hartree/Particle) of different transition states.
