# Supplementary material for: An Unusual Conformational Isomer of Verrucosidin Backbone from a Hydrothermal Vent Fungus, Penicillium sp. Y-50-10
Source: Mar Drugs. 2016 Aug 18;14(8):156. doi: 10.3390/md14080156 (PMC4999917; doi:10.3390/md14080156)
Supplement: Supplementary file 1 [file marinedrugs-14-00156-s001.pdf]

Supplementary Materials: An Unusual Conformational Isomer of Verrucosidin Backbone from a Hydrothermal Vent Fungus, *Penicillium* sp. Y-50-10

Chengqian Pan, Yutong Shi, Bibi Nazia Auckloo, Xuegang Chen, Chen-Tung Arthur Chen, Xinyi Tao and Bin Wu

Table of contents

Figure S1. ^{1}H NMR in CD_3OD for compound **1**.
Figure S2. ^{13}C NMR in CD_3OD for compound **1**.
Figure S3. DEPT in CD_3OD for compound **1**.
Figure S4. COSY in CD_3OD for compound **1**.
Figure S5. HMQC in CD_3OD for compound **1**.
Figure S6. HMBC in CD_3OD for compound **1**.
Figure S7. NOESY in CD_3OD for compound **1**.
Figure S8. HRESIMS for compound **1**.
Figure S9. B3LYP/6-31+G(d,p) calculated ECD data for conformations of compound **1**.
Figure S10. MS data (TIC and XIC $[M + H]^+$ 449 Da) for acetonitrile dissolved *Penicillium* sp. Y-50-10 extract.
Table S1. Gibbs Free Energy (Hartree/Particle) of compound **1**.
Table S2. Total Energy (Hartree/Particle) of different transition states.

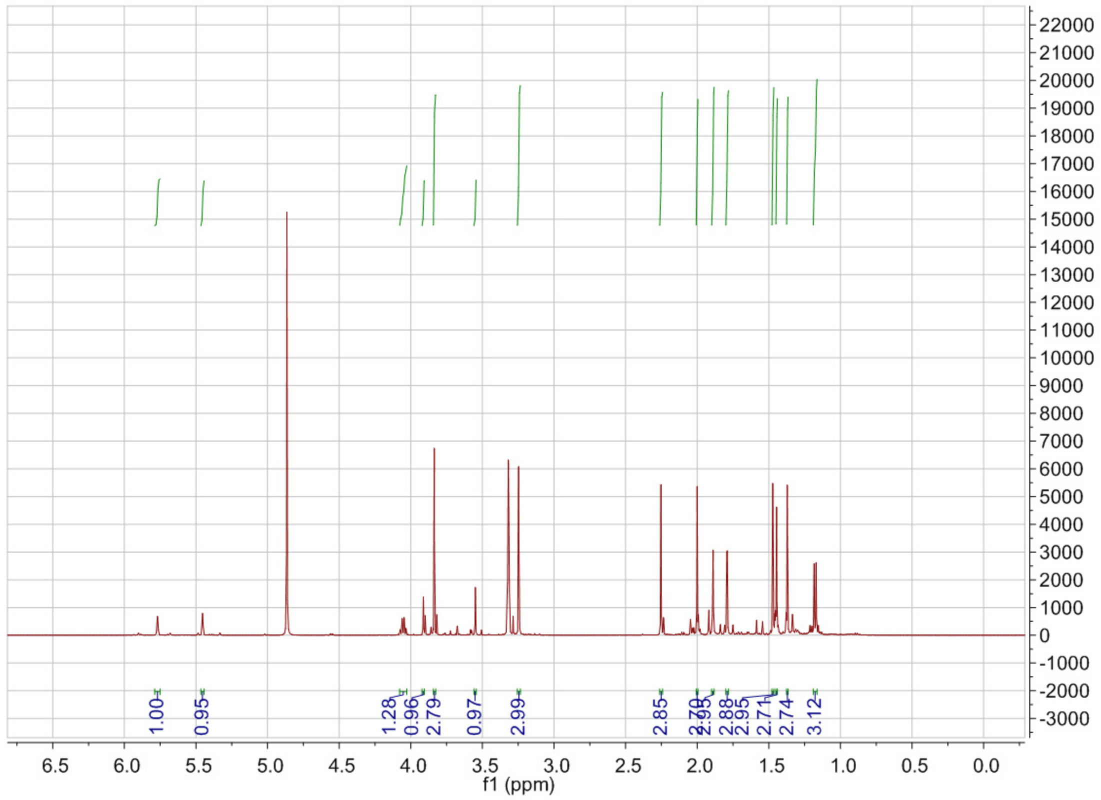

Figure S1. ^1H NMR in CD_3OD for compound **1**.

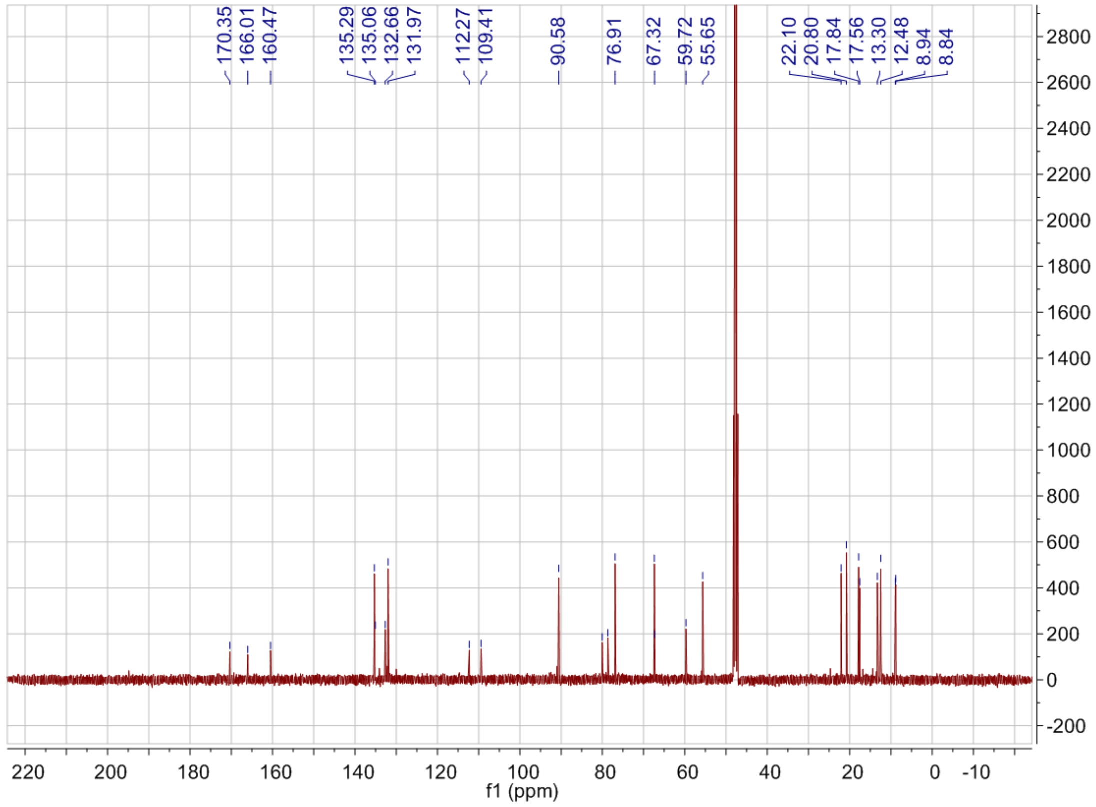

Figure S2. ^{13}C NMR in CD_3OD for compound **1**.

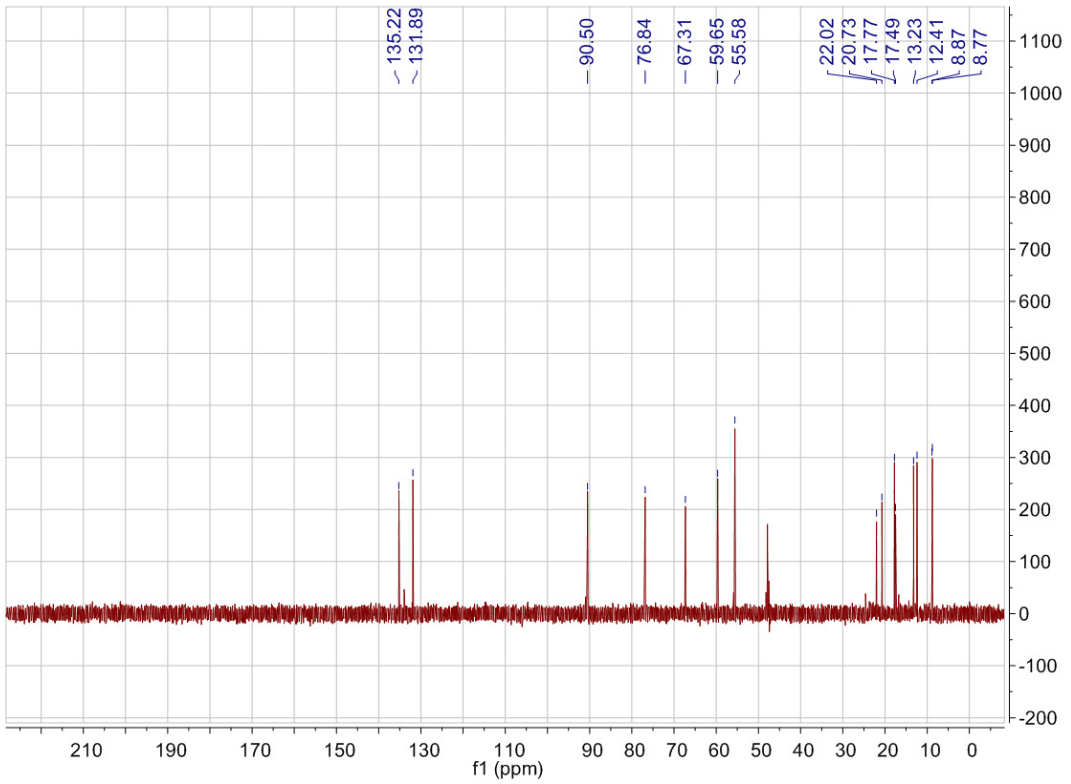

Figure S3. DEPT in CD_3OD for compound **1**.

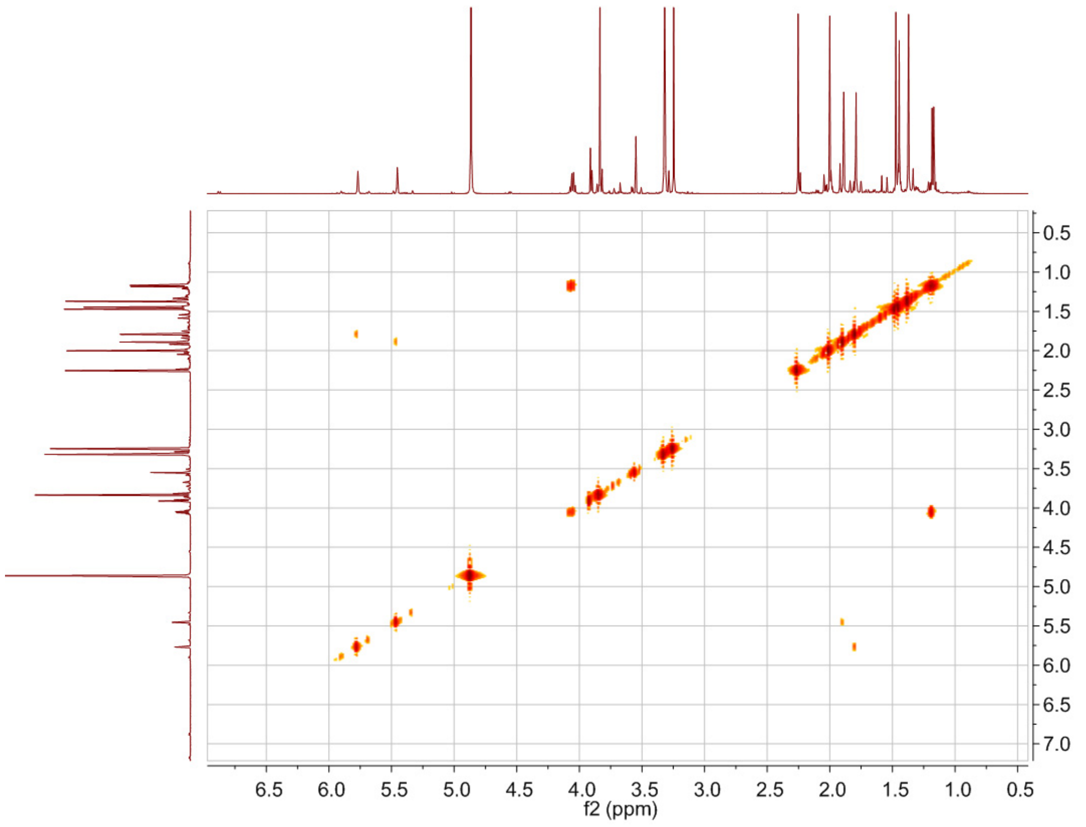

Figure S4. COSY in CD_3OD for compound **1**.

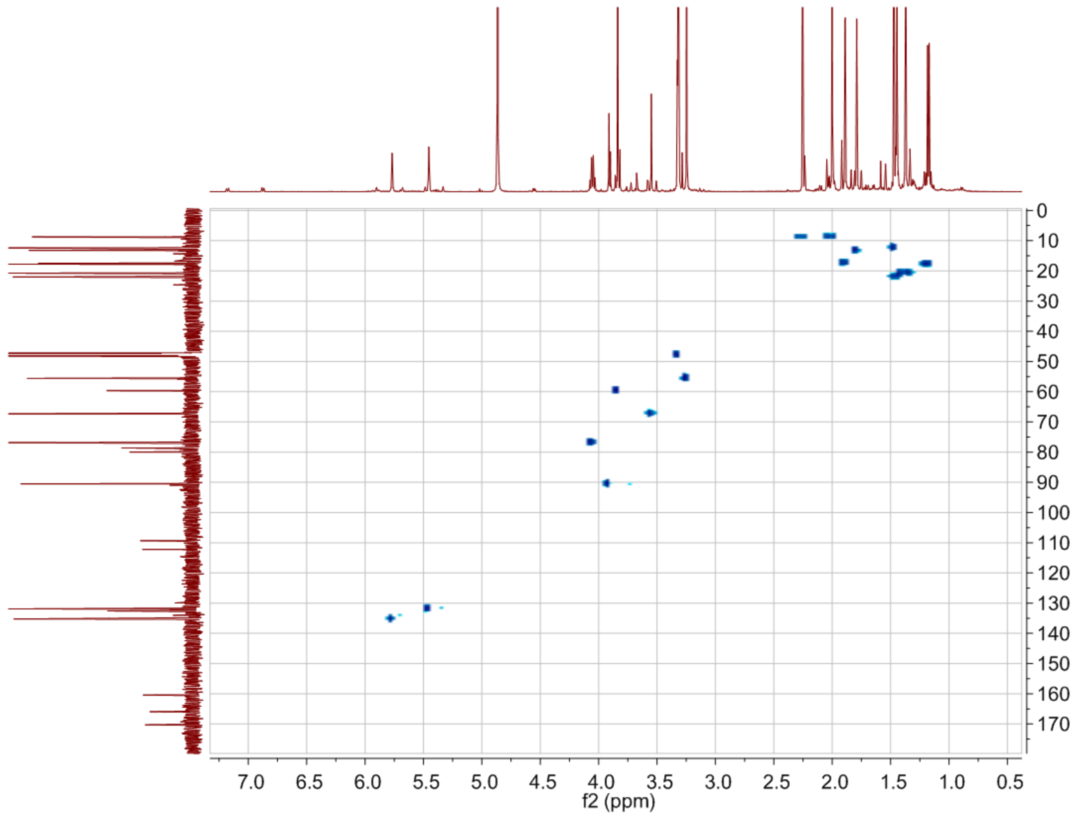

Figure S5. HMQC in CD_3OD for compound **1**.

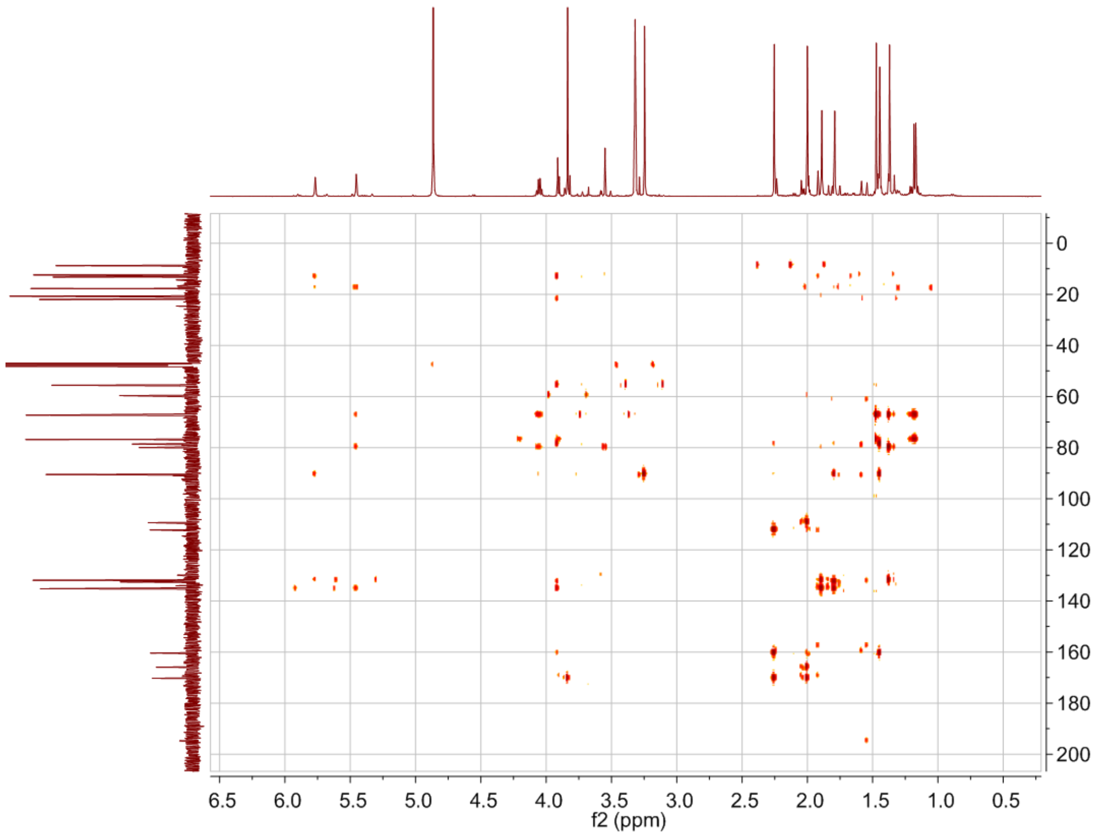

Figure S6. HMBC in CD_3OD for compound **1**.

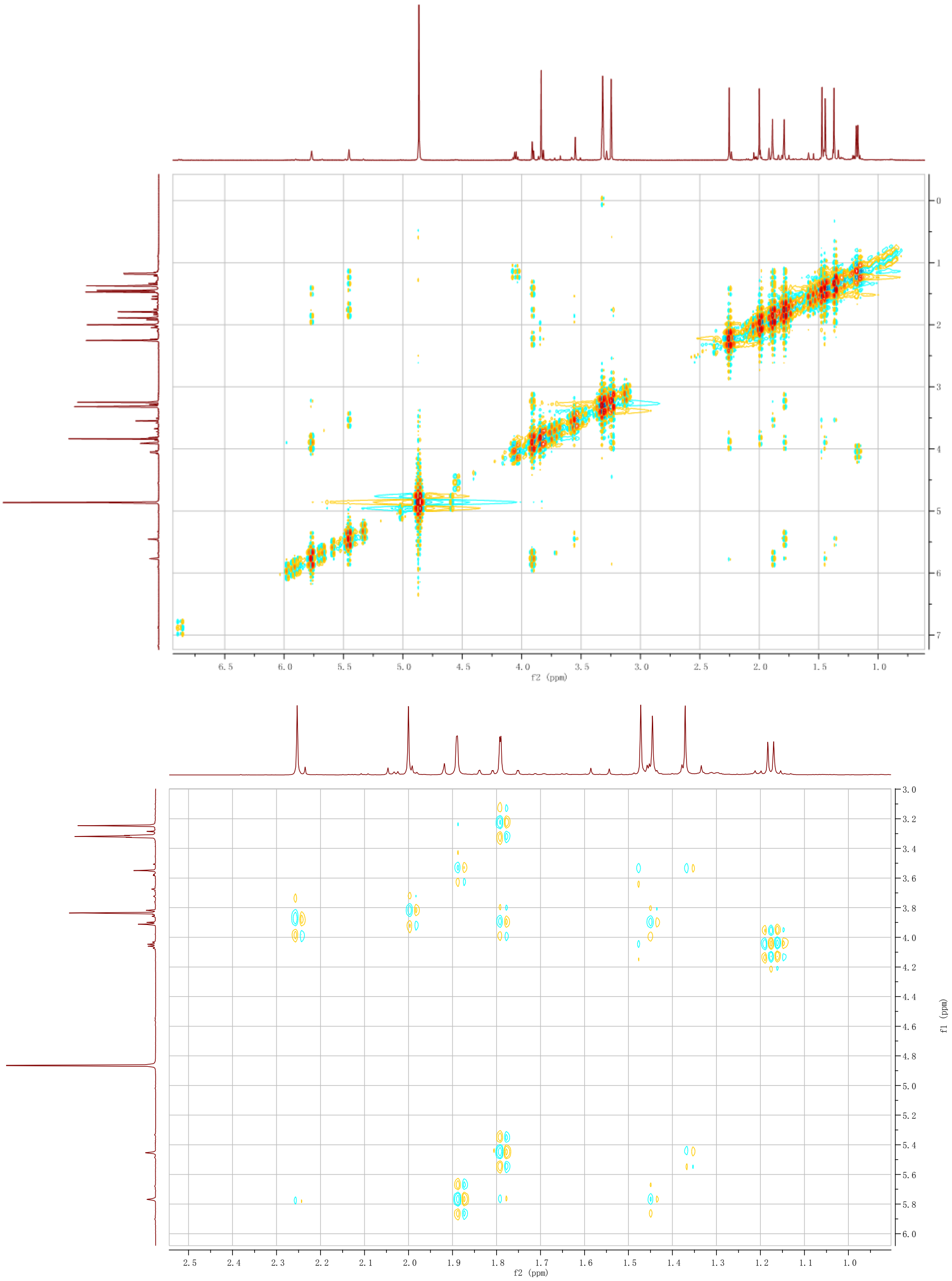

Figure S7. NOESY in CD_3OD for compound **1**.

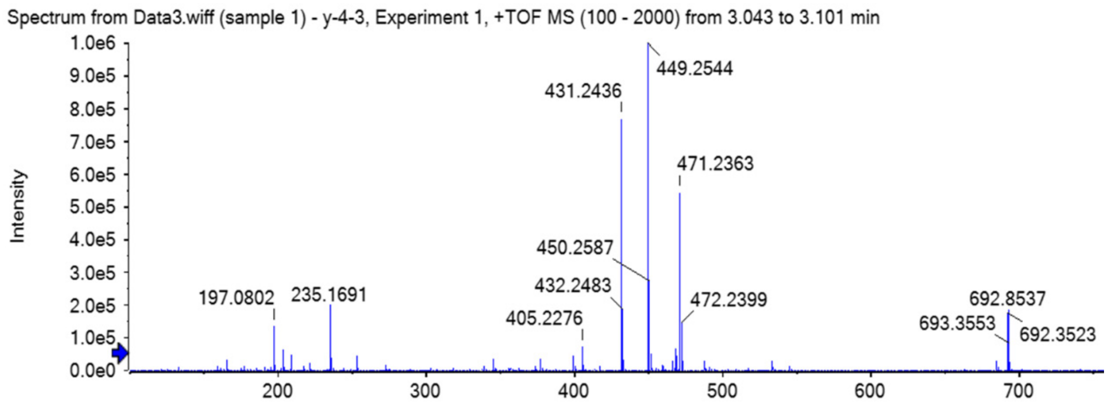

Figure S8. HRESIMS for compound **1**.

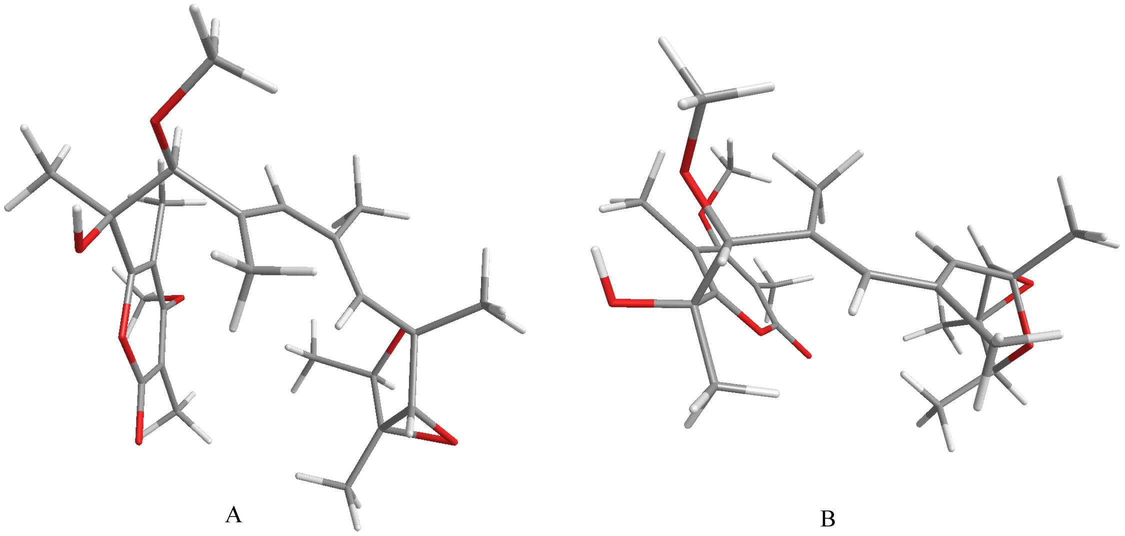

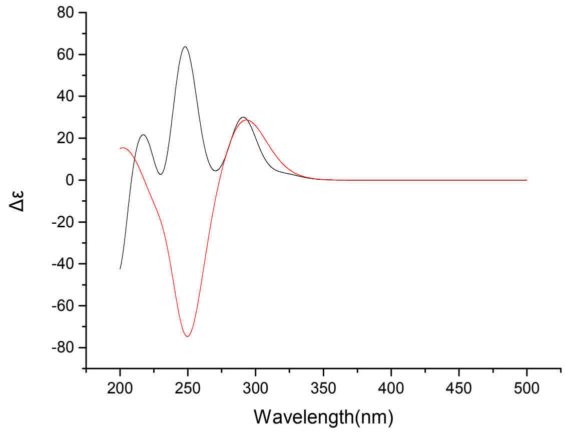

Figure S9. B3LYP/6-31+G(d,p) calculated ECD data for conformations of **A** (**red**) and **B** (**black**) of compound **1**.

TIC

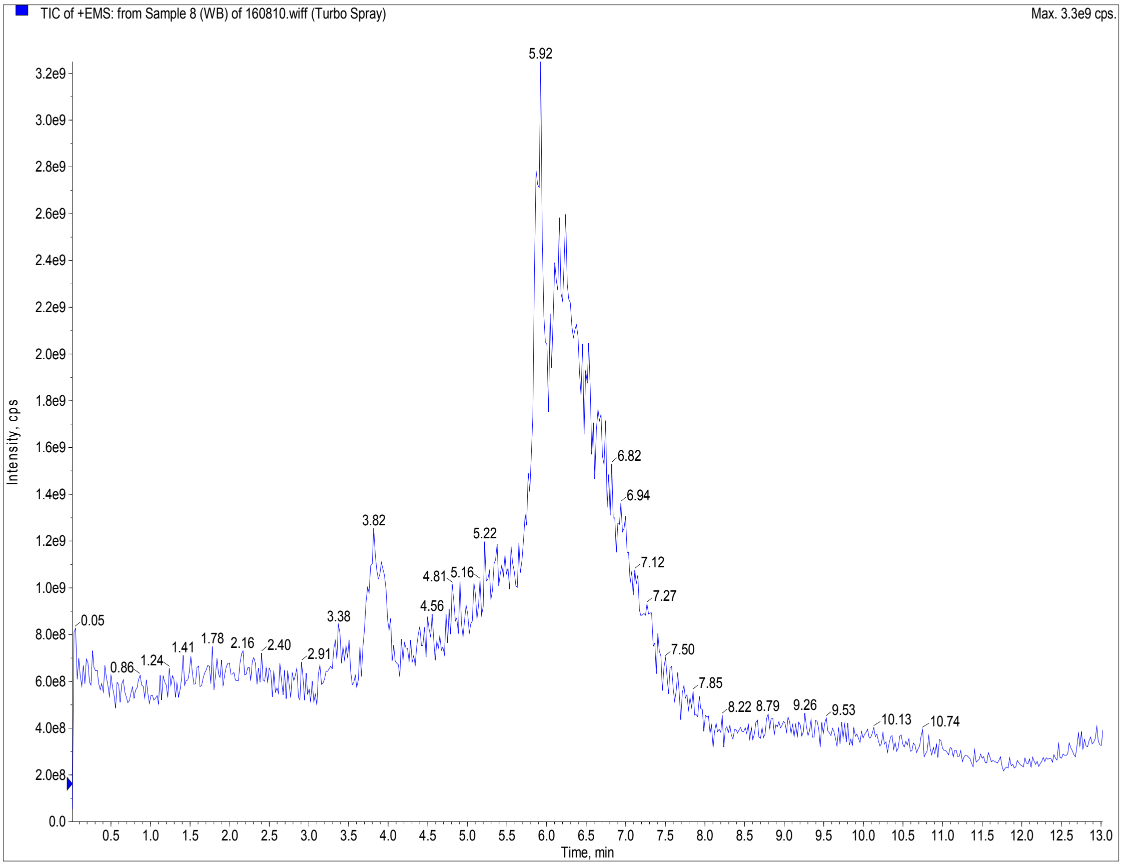

XIC [M + H]+ 449Da

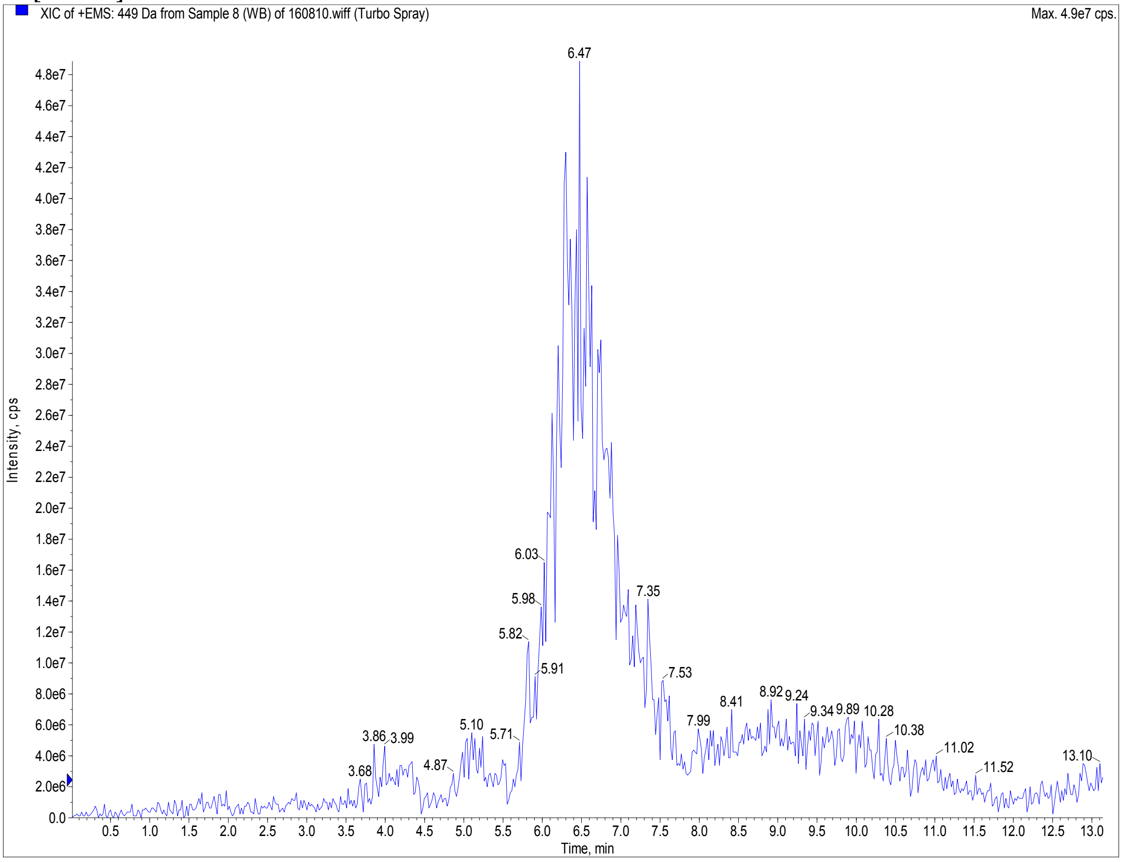

Figure S10. *Cont.*

6.47 min
$[M + H]^+$ 449
$[M + Na]^+$ 471

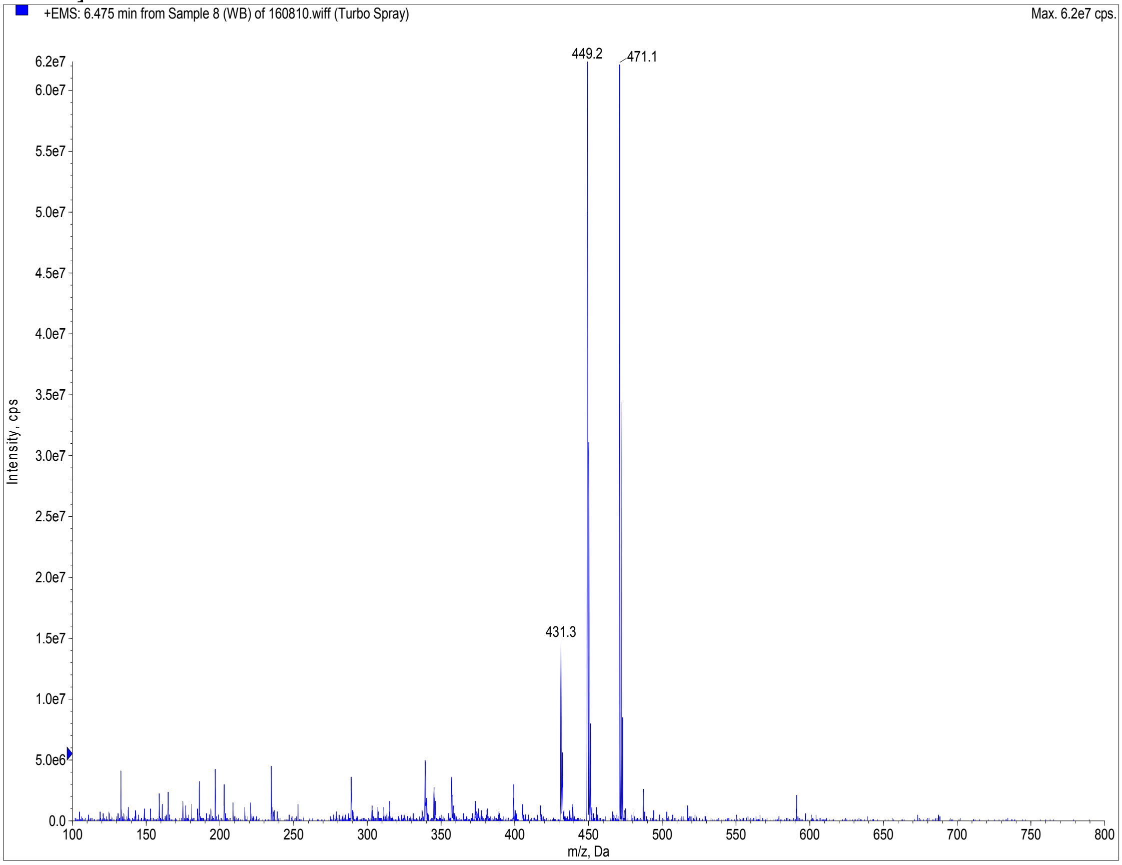

Figure S10. MS data (TIC and XIC $[M + H]^+$ 449 Da) for acetonitrile dissolved *Penicillium* sp. Y-50-10 extract.

Table S1. Gibbs Free Energy (Hartree/Particle) of compound **1**.

**Zero-Point Correction**	**0.577812 (Hartree/Particle)**
Thermal correction to Energy	0.608788
Thermal correction to Enthalpy	0.609732
Thermal correction to Gibbs Free Energy	0.516601
Sum of electronic and zero-point Energies	−1499.793597
Sum of electronic and thermal Energies	−1499.762620
Sum of electronic and thermal Enthalpies	−1499.761676
Sum of electronic and thermal Free Energies	−1499.854807

Table S2. Total Energy (Hartree/Particle) of different transition states.

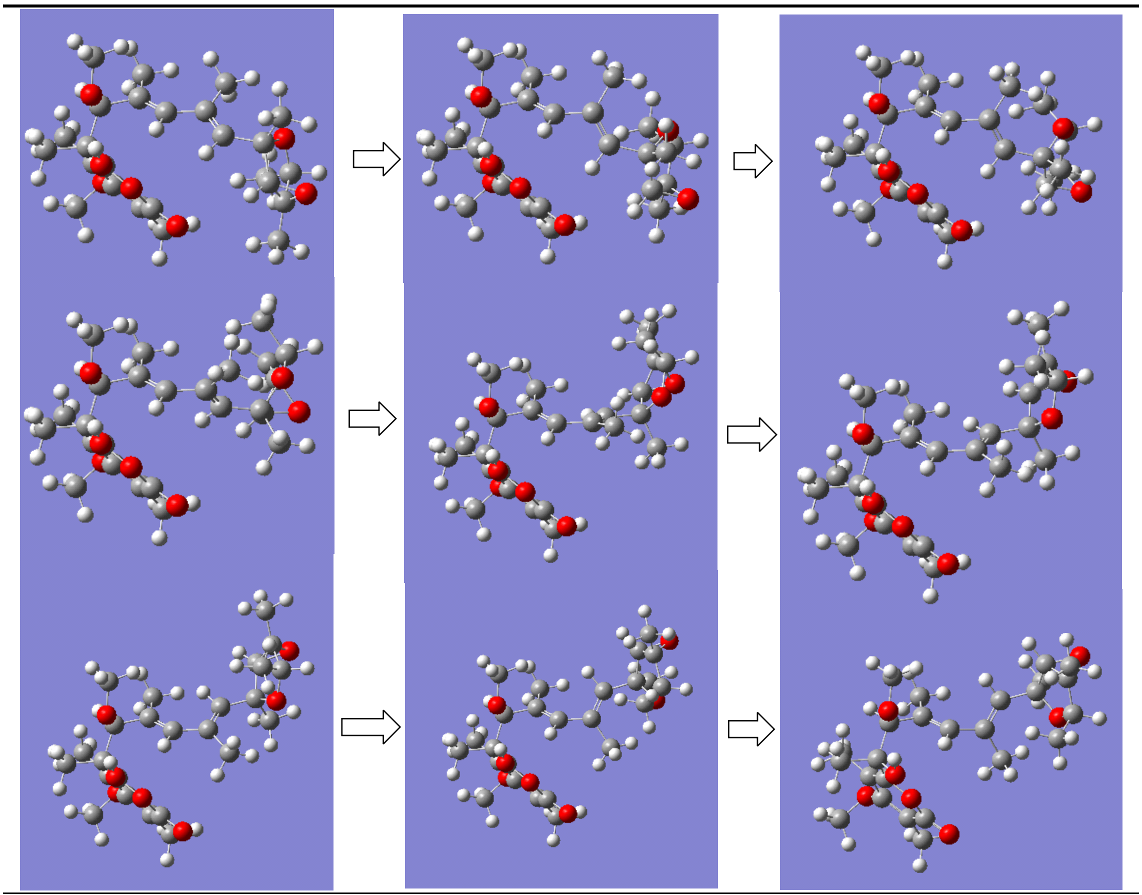

Scan of Total Energy	
# *X*-Axis: Scan Coordinate	# *Y*-Axis: Total Energy (Hartree)
−166.1186100000	−1500.3629599400
−146.1186100000	−1500.3615611600
−126.1186100000	−1500.3576511800
−106.1186100000	−1500.3554275500
−86.1186100000	−1500.3536596900
−66.1186100000	−1500.3554050000
−46.1186100000	−1500.3551308200
−26.1186100000	−1500.3517034100
−6.1186100000	−1500.3391024800
13.8813900000	−1500.3436973200
33.8813900000	−1500.3553624000
53.8813900000	−1500.3573404300
73.8813900000	−1500.3545908000
